# Cost Analysis of Three Techniques of Administering Sevoflurane

**DOI:** 10.1155/2014/459432

**Published:** 2014-10-29

**Authors:** Asha Tyagi, Vineeta Venkateswaran, Ajai Kumar Jain, Uttam Chandra Verma

**Affiliations:** Department of Anaesthesiology and Critical Care, University College of Medical Sciences and GTB Hospital, Shahdara, Delhi 110095, India

## Abstract

*Background*. This study aimed to evaluate and compare total cost of sevoflurane and propofol for 1.0 MAC-hour of anaesthesia, employing three anaesthetic techniques. *Methods*. Adult patients scheduled for surgical procedures under general anaesthesia anticipated to last approximately an hour were randomized into three groups (*n* = 15 each), to receive anaesthesia using one of the following techniques: low flow technique involving induction with propofol, followed by sevoflurane delivered using initial fresh gas flows of 6 L/min till MAC reached 1.0 and then reduced to 0.5 L/min; alternate method of low flow entailing only a difference in fresh gas flow rates being maintained at 1 L/min throughout; the third technique involving use of sevoflurane for both induction and maintenance of anaesthesia. *Results*. Cost of sevoflurane to maintain 1 MAC-hour of anaesthesia was clinically least with low flow anaesthesia, though statistically similar amongst the three techniques. Once the cost of propofol used for induction in two of the three groups was added to that of sevoflurane, cost incurred was least with the technique using sevoflurane both for induction and maintenance of anaesthesia, as compared to low flow and alternative low flow techniques, a 26% and 32% cost saving, respectively (*P* < 0.05).

## 1. Introduction

Newer inhalational agents such as sevoflurane offer several advantages over older agents but are comparatively more expensive. Evolving economic constraints across the world have brought forth the need to develop techniques that can minimize the cost of anaesthesia [1]. The technique of low flow anaesthesia (LFA) employing reduced fresh gas flow rates is known to decrease the consumption of inhalational agents such as sevoflurane and hence the cost of anaesthesia [2–5].

An “alternative method” of LFA has also been suggested to minimize the consumption of inhalational agent [6]. Herein reduced fresh gas flow rates are used right from the beginning and continued till the end of the anaesthetic procedure. This is in contrast to usual practice of LFA wherein fresh gas flow rates are reduced only after an initial period of higher flows [7]. However, this “alternative method of LFA” has not been evaluated when using sevoflurane as the inhalational agent.

In yet another anaesthetic technique, involving use of inhalational volatile agent for induction as well as maintenance of anaesthesia (VIMA) decreased cost is reported as compared to an intravenous induction followed by inhalational maintenance or continued use of the intravenous agent [8–10].

Thus, although these three anaesthetic techniques, namely, LFA, the alternative method of LFA, and VIMA, have each been documented to result in cost saving, there is no study comparing the costs of these three techniques with each other. The present investigation aimed to evaluate and compare the consumption and hence the cost of sevoflurane and propofol when used for induction with these techniques while maintaining 1 MAC concentration of the former.

As a secondary observation, the feasibility of administering sevoflurane with the “alternative method” of LFA was assessed, by measuring the time taken to attain 1.0 MAC of sevoflurane.

## 2. Materials and Methods

This randomized controlled trial was conducted after obtaining approval of the institutional ethics committee and written informed consent from all subjects.

Patients of either gender, aged between 18 and 60 years, belonging to ASA physical status I or II, and scheduled for short duration elective surgeries under general anaesthesia anticipated to last approximately one hour, were enrolled. Those with history of sensitivity to any of the drugs used in the study and in whom surgery lasted <30 minutes or >3 hours were excluded from the study.

A total of 45 patients were randomized using a computer generated random number table to receive one of three anaesthetic techniques using sevoflurane as the volatile inhalational agent (*n* = 15 each). The anaesthetic techniques included either LFA following induction with propofol or alternative method of LFA following induction with propofol or VIMA wherein both induction and maintenance were done using sevoflurane.

After shifting into the operating room, intravenous access was secured and monitoring including lead II electrocardiography, pulse oximetry, noninvasive oscillometric blood pressure measurements, and respiratory gas analysis was instituted (Penlon PM9000 Express). Fentanyl, 2 *μ*g/kg i.v., was administered to all patients prior to induction of anaesthesia. Subsequent anaesthetic technique was as per the group to which the patient was randomized.

### 2.1. Group L (Technique of LFA)

#### 2.1.1. Induction

Anaesthesia was induced with propofol 2-3 mg/kg i.v. titrated to loss of eyelash reflex and manual ventilation was initiated using a face-mask and rebreathing system with a carbon dioxide absorber, at a fresh gas flow rate of 6 L/minute (3-3 : oxygen-air) and a dial setting of 5% on sevoflurane vaporizer. On achieving a MAC value of 1.0 on the respiratory gas monitor, the flow rate was reduced to 0.5 L/minute (0.3-0.2 : oxygen-air).

#### 2.1.2. Intubation

Rocuronium 0.6 mg/kg i.v. was administered after induction of anaesthesia with propofol and intubation was performed three minutes later, provided 1.0 MAC of sevoflurane had been attained. If the MAC of sevoflurane at this time was <1.0, manual ventilation was continued for another two minutes with sevoflurane titrated to attain the desired MAC. If MAC did not rise to 1.0 even after this additional time being allowed, one-fifth of the induction dose of propofol was administered and tracheal intubation was performed.

#### 2.1.3. Maintenance of Anaesthesia

For titrating the intraoperative MAC to 1 ± 0.2, the dial setting of the sevoflurane vaporizer was increased or decreased by 0.5% at intervals of two minutes. Intraoperative mechanical ventilation was initiated with a tidal volume of 8 mL/kg and a respiratory rate of 10 breaths/minute, with the rate titrated to maintain end-tidal carbon dioxide of 35 to 40 mmHg.

### 2.2. Group A (Technique of Alternative Method of LFA)

#### 2.2.1. Induction

As compared to Group L, the only differences during induction were a reduced fresh gas flow rate of 1 L/minute as compared to 6 L/minute and higher output of sevoflurane vaporizer set at 8% as compared to 5%. The fresh gas flow rate was maintained at 1 L/minute even after achieving a MAC value of 1.0.

Protocols for intubation, sevoflurane vaporizer setting during maintenance of anaesthesia, and intraoperative ventilation were similar to those in Group L.

### 2.3. Group V (Technique of VIMA)

#### 2.3.1. Induction

Previously documented method of vital capacity rapid induction (VCRI) with sevoflurane was used [11, 12]. Prior to induction of anaesthesia, the patient practiced the VCRI maneuver with the anaesthetist, namely, to exhale fully and then inhale fully and hold his/her breath as long as possible. The breathing system was primed after occluding its patient-end and running a flow rate of 6 L/minute (3-3 : oxygen-air) with the sevoflurane vaporizer set at 8% for 60 seconds. After 60 seconds, the gas flows were turned off with the patient-end of the breathing system still occluded. The patient was then instructed to take a maximal inspiratory breath through the primed circuit with gas flow turned on at 6 L/minute (3-3 : oxygen-air) and sevoflurane vaporizer set at 8%, holding his/her breath for as long as possible. Extra vital capacity breaths were taken if necessary. After loss of eyelash reflex, the rate was reduced to 0.5 L/minute (0.3-0.2 : oxygen-air) with the sevoflurane dial setting sufficient to maintain a MAC value of 1 ± 0.2.

Protocols for intubation, sevoflurane vaporizer setting during maintenance of anaesthesia, and intraoperative ventilation were followed as in both of the other groups.

In all three groups, fentanyl was used to supplement analgesia intraoperatively. At the end of the operative procedure and at the start of skin closure, the vaporizer was turned off and lungs were ventilated with oxygen at 6 L/minute. Residual neuromuscular paralysis was reversed with neostigmine 0.5 mg/kg i.v. along with glycopyrrolate 0.2 mg/kg i.v.

Due to the nature of the anaesthetic procedure, that is, intravenous versus inhalational induction, it was not possible to blind the patient or the anaesthetist. However, the primary outcome measure, namely, cost of sevoflurane and propofol, was estimated from their consumption that was judged by objective parameters.

### 2.4. Recorded Parameters

The consumption of sevoflurane (Sevorane; Abbott Laboratories, USA) for each patient was calculated from the difference in weight of the vaporizer before and after the anaesthetic procedure [3, 13–15], using a precision weighing scale with least count of 0.1 gm (PCB 10000-1; Kern Germany). The weight of the vaporizer (Penlon Sigma Delta, Penlon, UK) on both occasions was calculated from an average of three consecutive readings. The difference in weight (gm) obtained was divided by the density of sevoflurane to calculate the consumption of sevoflurane in mL [16]. Consumption of sevoflurane (mL) was converted to “MAC-hour” consumption by dividing it by the entire duration of 1.0 MAC anaesthesia. Cost of sevoflurane for “MAC-hour” consumption was derived using market retail price of sevoflurane [Indian Rupee (INR)*₹* 8500 for 250 mL].

The cost of propofol (Neorof; Neon Laboratories Limited, India) used for induction in Groups L and A was calculated for the entire vials dispensed, that is, drug actually used for induction along with the wasted unused fraction left in the opened vial. The market retail price of the 20 mL vial of propofol was used for calculation of cost (INR*₹* 198/vial).

The cost of propofol was added to the “MAC-hour cost” of sevoflurane to derive the total MAC-hour cost for comparison of cost between the three groups.

Time to attain 1.0 MAC of sevoflurane was calculated as starting from the time when the face-mask attached to the primed (Group V) or unprimed (Groups L and A) breathing system was placed on the patient's face to time of attainment of 1.0 MAC on respiratory gas monitor.

To allow for comparison of the three study groups, demographic characteristics including age, gender, weight of the patient, and intraoperative characteristics such as duration of surgery (incision to closure), duration of anaesthesia (vaporizer on to vaporizer off), and pre- and postintubation heart rate and mean arterial pressure were also recorded. The inspired (*F*_*I*_) and end-tidal (*F*_*E*_) concentrations and MAC of sevoflurane were noted every minute for the first five minutes or till intubation, whichever was earlier, followed by every five minutes after intubation till attainment of 1 ± 0.2 MAC, and then every 15 minutes till the end of anaesthesia.

### 2.5. Statistical Analysis

One-way ANOVA followed by Tukey's test or Dunnett's T3 was employed for comparison of quantitative data amongst the three groups. Nominal data was analyzed using chi-square test. The level of significance was taken as <0.05 for the study.

### 2.6. Sample Size

There is no previous study comparing the three techniques of anaesthesia. We decided to enroll a minimum of 15 patients in each group, with an interim analysis done at 50% of completion of enrollment. This revealed a pooled standard deviation of 90 for the total cost of sevoflurane and propofol for 1 MAC-hour of anaesthesia. To detect a 20% difference in cost amongst the three groups at 80% power and *α* error of 5%, a minimum of 13 patients were required in each group. To accommodate for possible loss to successful conduct of the protocolized anaesthetic management, 15 patients were enrolled in each group.

## 3. Results

There was no statistically significant difference with respect to the demographic parameters amongst the three groups (Table 1). The mean durations of surgery and anaesthesia were also statistically similar amongst the three groups, though clinically shorter with Group L as compared to both of the other groups (Table 2). Time to attain 1.0 MAC of sevoflurane was significantly shorter for Group V as compared to both Groups L and A (*P* = 0.000), as well as for Group L as compared to Group A (*P* = 0.017) (Table 2). The number of patients attaining a MAC of 1.0 at the proposed time of intubation, that is, three minutes after the injection of rocuronium, was 15/15 (100%), 12/15 (80%), and 7/15 (47%) in Group V, Group L, and Group A, respectively. In all remaining patients of Groups L and A (3/15 and 8/15, resp.), 1.0 MAC was attained after waiting for an additional two minutes. The duration of 1.0 MAC anaesthesia was statistically similar amongst the three groups (*P* = 0.575).

The difference between preinduction and postintubation heart rate as well as mean arterial pressure was statistically similar amongst the three groups (Table 2).

The consumption of sevoflurane for the entire duration of anaesthesia was clinically higher in Groups A and V as compared to Group L but remained statistically similar amongst the three groups (Table 3). The consumption as well as cost of sevoflurane for an hour of 1.0 MAC anaesthesia, negating the effect of duration of anaesthesia, was statistically similar amongst all groups (Table 3). Amount of propofol consumed for induction in Groups L and A was statistically similar (*P* = 0.867) (Table 3). The cost of propofol calculated for either actual amount used or the vials procured per patient was similar in both groups in which it was used, namely, Groups L and A (*P* = 1.000) (Table 3).

The total cost of sevoflurane and propofol together, for MAC-hour of anaesthesia, was significantly lesser with Group V as compared to Groups L and A (*P* = 0.000), with no significant difference between Group L and Group A (*P* = 0.893) (Table 3).

**Figure 1:**
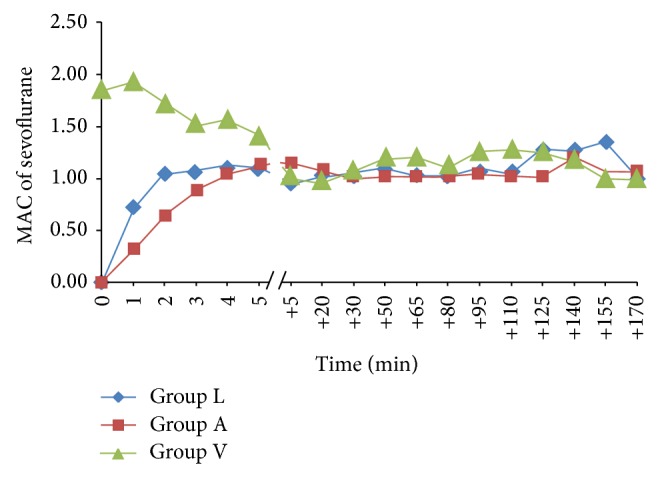
MAC of sevoflurane versus time. Group L: low flow anaesthesia, Group A: alternative method of low flow anaesthesia, and Group V: volatile induction and maintenance of anaesthesia.

**Figure 2:**
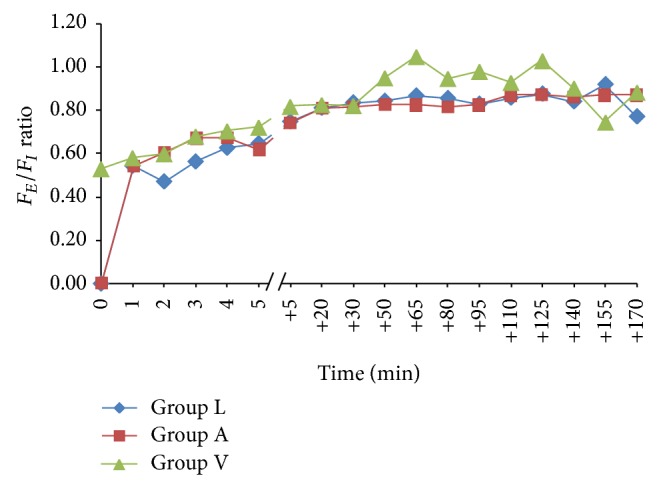
*F*_*E*_/*F*_*I*_ ratio versus time. *F*_*E*_: end-tidal concentration of sevoflurane and *F*_*I*_: inspired concentration of sevoflurane; Group L: low flow anaesthesia, Group A = alternative method of low flow anaesthesia, and Group V = volatile induction and maintenance of anaesthesia.

Figure 1 shows the intraoperative MAC of sevoflurane in the three groups. At the time of induction (time = 0; Figure 1) the MAC of sevoflurane was zero in Groups L and A since anaesthesia was induced using intravenous agent, with consequent lack of sevoflurane in the fresh gas mixture. In contrast, MAC of approximately 2.0 was observed for Group V at this time since the breathing system was primed prior to induction (time = 0; Figure 1). Following induction, intraoperative MAC of 1 ± 0.2 was maintained in all three groups by titrating the sevoflurane output.  Figure 2 shows the *F*_*E*_/*F*_*I*_ curves, representing ratio of end-tidal to inspired concentration for sevoflurane, for each of the three groups.

## 4. Discussion

It was observed that consumption and hence the cost of sevoflurane were clinically lesser though statistically similar to LFA as compared to the other two techniques. However, once the cost of propofol used for induction with LFA and its alternative method was added to the cost of sevoflurane, VIMA was the most cost-effective of the three anaesthetic techniques. The MAC-hour cost for VIMA was significantly less as compared to LFA and the alternative method of LFA techniques (INR*₹*  373 ± 125, 506 ± 53, and 552 ± 71, resp.). This cost difference translates to 26% or 32% cost saving in the mean MAC-hour cost with VIMA as compared to LFA and alternative method of LFA, respectively (*P* < 0.05). When considered in terms of total savings for a particular institute over months or a year, the amount can be expected to be substantial.

Previously also, the cost of VIMA using sevoflurane was noted to be cheaper as compared to induction with propofol followed by maintenance with sevoflurane [17, 18] or induction as well as maintenance with propofol [8, 9]. Studies that have compared propofol and sevoflurane for induction only have also noted lower costs with sevoflurane induction [10].

While the technique of LFA is a well-known and commonly applied anaesthetic practice, use of alternative method of LFA and VIMA is comparatively less common. VIMA was associated with rapid attainment of 1.0 MAC with mean time of 0.6 ± 0.6 minutes during induction and high intraoperative *F*_*E*_/*F*_*I*_ values (approximating 1.0). This implies the clinical feasibility of using sevoflurane for inhalation induction and maintenance of anaesthesia, suggesting that VIMA can be used as an acceptable clinical practice with the intention of cost-saving. The technique of alternative method of LFA also allowed attainment of 1.0 MAC in a mean time of 3.5 ± 1.1 minutes with a maximum of 5 minutes and thus can be applied in clinical practice, though it remains significantly more expensive than VIMA and LFA.

The technique of LFA involves an initial period of higher fresh gas flow rates aiming to denitrogenate the breathing system and patient's lungs and accommodate the initial rapid uptake of inhalational agent so as to rapidly build the alveolar (represented by end-tidal) concentrations [7]. This period of initial higher flow rates varies from short periods terminating once airway is secured [3, 13] to longer period of even 20 minutes following intubation [7]. We used fresh gas flow at initial rate of 6 L/minute till 1.0 MAC of sevoflurane was attained and then decreased to 0.5 L/minute since it is an acceptable and desirable target for anaesthesia [19]. Even with the alternative method of LFA, the use of decreased flow since the beginning did not interfere with attainment of early increase in sevoflurane concentration. The mean time to achieve 1.0 MAC with LFA was 2.5 ± 1.2 minutes with a range of 1 to 5 minutes and with alternative method an average of 3.5 ± 1.1 minutes with a range of 2 to 5 minutes.

During VIMA, induction can be carried out with the patient taking tidal volume or vital capacity breaths, from an unprimed or primed breathing system [8, 11, 12, 20]. Vital capacity breathing results in a quicker induction than tidal volume breathing [12] and hence is appropriately termed as a “rapid induction” [12, 20]. The technique of priming the breathing system for a VCRI technique has varied in previous studies. A 30-second period was noted to be adequate for priming the breathing system when using 100% oxygen at a flow rate of 8 L/minute and sevoflurane vaporizer output of 8% [21]. We primed the breathing system for a longer duration of 60 seconds using mixture of oxygen-air at a slightly lower flow rate of 6 L/minute and sevoflurane vaporizer setting of 8%. The adequacy of the induction method used in our study is evident from the MAC value of 1.5 for sevoflurane achieved at the beginning of induction itself.

There was no significant difference in heart rate or mean arterial pressure changes following intubation amongst the three techniques. Previously also, the technique of VIMA was associated with similar hemodynamic consequences as compared to intravenous induction with propofol followed by maintenance of anaesthesia with sevoflurane [10, 11].

Postoperative renal function tests were not done in this study. The concern of potential nephrotoxicity due to formation of compound A (fluoromethyl-2-2-difluoro-1-trifluoromethyl vinyl ether) following use of sevoflurane with decreased fresh gas flow rates [22] has now been negated in several clinical studies [23–26]. Clinical practice guidelines also no longer appear to impose restrictions on use of sevoflurane with reduced fresh gas flow rates [27], and to date no significant clinical renal toxicity has been associated with the use of sevoflurane [28].

This study does not take into account the costs of anaesthesia related to other agents such as the fresh gas flow mixture. Taking into consideration the rates of fresh gas flow mixture used with LFA, alternative method of LFA, and VIMA, respectively, in our study, its total consumption for the first hour of anaesthesia would be 45 L, 60 L, and 39 L with the three techniques, respectively. Since the volume of fresh gas flow is the least with VIMA, even if its cost were to be added to that of sevoflurane and proposal, VIMA would likely remain the least expensive technique. The results of this study are derived from and thus are applicable to short-duration surgeries only. The cost analysis may change if applied to surgeries of shorter or more prolonged duration since the uptake of sevoflurane is a time-dependent phenomenon. Given the low solubility of sevoflurane and minimal uptake by tissues, the precise variations in cost analysis with respect to changes in duration of anaesthesia may be small.

Lastly, in our study, the cost analysis pertains to the consumption of sevoflurane and propofol only, when using three different anaesthetic techniques. Addition of the cost of disposables, other drugs used to counter clinical side effects of sevoflurane or propofol, or the implications of these drugs in the duration of hospitalization-related expenses have not been considered.

Based on our observations, we recommend the use of VIMA as a cost-saving, effective technique for adult patients scheduled for short-duration surgeries under general anaesthesia.

## Figures and Tables

**Table 1:** Patient characteristics.

Characteristics	Group L (*n* = 15)	Group A (*n* = 15)	Group V (*n* = 15)	*P* value
Age (years)	39 ± 9	37 ± 9	33 ± 10	0.231
Weight (kg)	61 ± 11	58 ± 15	63 ± 14	0.559
Gender (M : F)	5 : 10	2 : 13	6 : 9	0.245
ASA physical status (I : II)	11 : 4	11 : 4	14 : 1	0.287

Data presented as mean ± standard deviation or number of patients. Group L: low flow anaesthesia; Group A: alternative method of low flow anaesthesia; Group V: volatile induction and maintenance of anaesthesia; M: male; F: female; ASA = American Society of Anesthesiologists.

**Table 2:** Intraoperative characteristics.

Characteristics	Group L (*n* = 15)	Group A (*n* = 15)	Group V (*n* = 15)	*P* value
Duration of surgery (min)	99 ± 40.6	119.7 ± 47.3	126.8 ± 51.7	0.250
Duration of anaesthesia (min)	110.1 ± 43.8	133.2 ± 54.3	129.5 ± 48.3	0.392
Time to attain 1.0 MAC (min)	2.5 ± 1.2	3.5 ± 1.1^Φ^	0.6 ± 0.6^†#^	0.000
Duration of 1.0 MAC anaesthesia (min)	107.4 ± 44.2	134.1 ± 52.6	126.8 ± 51.7	0.575
HR difference^*^	−1.9 ± 13.2	−5.5 ± 22.2	−12 ± 27.8	0.449
MAP difference^*^ (mmHg)	6.4 ± 16.8	5.9 ± 9.4	2.6 ± 20.9	0.787

Data presented as mean ± standard deviation. Group L: low flow anaesthesia; Group A: alternative method of low flow anaesthesia; Group V: volatile induction and maintenance of anaesthesia; HR: heart rate; MAP: mean arterial pressure; MAC: minimum alveolar concentration. ^*^Significant difference between preinduction and postintubation parameters; *P* < 0.05 for ^Φ^Group A versus Group L, ^†^Group V versus Group L, and ^#^Group V versus Group A.

**Table 3:** Consumption and cost of sevoflurane and propofol.

Characteristics	Group L (*n* = 15)	Group A (*n* = 15)	Group V (*n* = 15)	*P* value
Sevoflurane consumed (gms)	25.7 ± 11.2	33.4 ± 15.3	33.3 ± 10.2	0.168
Sevoflurane consumed (ml)	16 ± 5	22.6 ± 10.8	21.9 ± 6.7	0.098
Sevoflurane consumed/MAC-hour (ml)	9 ± 1.5	10.4 ± 2.1	10.9 ± 3.7	0.133
Cost of sevoflurane/MAC-hour (INR)	308 ± 53	354 ± 71	373 ± 125	0.197
Propofol consumed (mg)	120 ± 26	118 ± 25	Not applicable	0.867
Cost of propofol used (INR)	119 ± 25	117 ± 25	Not applicable	0.858
Cost of propofol vial dispensed^*^ (INR)	198 ± 0	198 ± 0	Not applicable	1.000
Cost of anaesthesia/MAC-hour (INR)	506 ± 53	552 ± 71	373 ± 125^†^	<0.001

Data presented as mean ± standard deviation. Group L: low flow anaesthesia; Group A: alternative method of low flow anaesthesia; Group V: volatile induction and maintenance of anaesthesia; MAC: minimum alveolar concentration. ^*^Calculated by the difference in weight of the vaporizer before and after anaesthesia; ^*^cost includes cost of the used and the unused wasted propofol, that is, entire vial opened for use in one patient. ^†^ *P* < 0.05 for Group V versus Group L as well as Group A.
